# Risk of Tumor Progression after Microsurgery for Parasellar Meningioma Invading the Cavernous Sinus

**DOI:** 10.3390/cancers16122217

**Published:** 2024-06-14

**Authors:** Arkadiusz Nowak, Edyta Maj, Andrzej Marchel, Przemysław Kunert

**Affiliations:** 1 Department of Neurosurgery, Medical University of Warsaw, 02-097 Warsaw, Poland; andrzej.marchel@wum.edu.pl (A.M.); pkunert@wp.pl (P.K.); 2 2nd Department of Clinical Radiology, Medical University of Warsaw, 02-097 Warsaw, Poland; em26@wp.pl

**Keywords:** parasellar meningioma, neurosurgery, postoperative radiotherapy, progression-free survival, cavernous sinus

## Abstract

**Simple Summary:**

Parasellar meningiomas are rare tumours of the skull base that may invade the cavernous sinus, making complete removal of the tumour impossible. The remaining tumour remnant is observed or treated with radiotherapy. This study aimed to assess which prognostic factors influence progression-free survival (PFS) in cases of incomplete removal of parasellar meningioma invading the cavernous sinus. The study included 32 patients who underwent surgery owing to parasellar meningioma invading the cavernous sinus. In some cases, the tumour was almost completely removed (near-total resection, NTR), with the tumour remnant left in the cavernous sinus, while in the remaining patients, the tumour was subtotally removed, leaving a small extracavernous portion (subtotal resection, STR). We found that radiotherapy only influenced PFS in the group that underwent STR, and tumour size was an independent factor affecting PFS after surgery. In conclusion, we found that a tumour remnant left in the cavernous sinus can be safely observed, but leaving an extracavernous tumour fragment in place requires adjuvant radiotherapy or radiosurgery to achieve meningioma control.

**Abstract:**

Background: Parasellar meningiomas, which may invade the cavernous sinus, pose a significant challenge to neurosurgeons due to the high risk of postoperative neurological deficits associated with aggressive resection of the intracavernous part of the tumour. Therefore, subtotal tumour removal followed by observation or radiotherapy for the residual meningioma in the cavernous sinus is recommended. This retrospective study aimed to identify prognostic factors influencing recurrence and progression-free survival (PFS) in parasellar meningiomas invading the cavernous sinus after incomplete surgical treatment. Methods: This study included adult patients diagnosed with benign parasellar meningioma (WHO Grade I) invading the cavernous sinus, treated at our institution between 2006 and 2020, and with a postsurgical follow-up of at least 3 years. Surgical treatment involved near-total resection (NTR) with an intracavernous residual tumour or subtotal resection (STR) with additional extracavernous tumour left in place. Kaplan–Meier analysis estimated PFS rates, and Cox regression tested survival time differences between groups. Results: Among the 32 patients, the estimated median PFS was 11 years. Radiotherapy improved 5-year PFS only in patients with STR (*p* = 0.003). The univariate analysis identified preoperative tumour size, low preoperative Karnofsky Performance Score (KPS), and marked brain oedema as significant factors affecting meningioma progression after surgery. The multivariate analysis confirmed tumour size as an independent factor for progression (*p* = 0.012). Conclusions: For patients with parasellar meningioma invading the cavernous sinus, extracavernous tumour removal followed by close radiological surveillance of the residual intracavernous meningioma is a safe and appropriate strategy. When an extracavernous tumour component is left, adjuvant stereotactic radiotherapy or radiosurgery is recommended to control tumour growth.

## 1. Introduction

Intracranial meningiomas arise from the cerebral meninges and typically have benign histological features. They are the most common non-malignant intracranial tumours, accounting for up to one-third of all primary central nervous system tumours [1]. Within meningiomas, parasellar meningiomas pose a significant challenge to neurosurgeons because of their involvement in major neurovascular structures and the possible invasion of the cavernous sinus. These tumours are divided according to their anatomical location on the skull base and the involvement of neighbouring structures into the medial sphenoid wing, tentorial incisura, and sphenopetroclival meningiomas and meningiomas confined to the cavernous sinus [2]. Cavernous sinus involvement is one of the major factors influencing the extent of surgical resection in parasellar meningiomas. Aggressively resecting a meningioma invading the cavernous sinus carries a high risk of additional neurological deficits, as well as the risk of death. Therefore, surgery is recommended for the meningioma extracavernous portion, with subsequent radiotherapy for the tumour remnant left in the cavernous sinus. Moreover, parasellar meningiomas often surround critical neurovascular structures on the skull base; thus, to ensure good functional outcomes, partial meningioma resection may be necessary, leaving behind not only an intracavernous piece of tumour but also a residual extracavernous tumour.

At our institution, parasellar meningiomas invading the cavernous sinus are generally treated using subtotal microsurgical resection, followed by adjuvant radiotherapy (stereotactic radiosurgery or fractionated stereotactic radiotherapy) or simple observation over time. The purpose of this retrospective study was to determine prognostic factors that may affect the further growth of parasellar meningiomas invading the cavernous sinus after incomplete surgical treatment. In particular, we aimed to investigate the impact of the extent of microsurgical resection and the use of postoperative radiotherapy on progression-free survival in these tumours. In addition, we aimed to analyse and compare functional outcomes and surgical results regarding the extent of microsurgical resection and other clinical factors.

## 2. Materials and Methods

### 2.1. Patient Selection

We reviewed all adult patients treated surgically at our institution between 2006 and 2020 for intracranial meningiomas with cavernous sinus tumour invasion. Parasellar meningiomas with cavernous sinus invasion were defined as skull-base meningiomas (petroclival, tentorial, and sphenoid wing meningiomas) that invaded the cavernous sinus or tumours with a predominant cavernous sinus component. Identification of the tumour was based on preoperative MRI.

### 2.2. Patient Population

We included patients with a diagnosis of parasellar meningioma invading the cavernous sinus who had undergone meningioma resection with a postsurgical follow-up of at least 3 years. Exclusion criteria included the following characteristics of eligible individuals: (1) patients harbouring multiple meningiomas, (2) WHO Grade II or III meningioma, (3) history of cancer, (4) neurofibromatosis or other genetic predispositions, (5) prior surgery or radiotherapy for the tumour.

### 2.3. Surgical Approach

Medial sphenoid wing meningiomas were mostly operated on via middle fossa approaches, including pterional craniotomy and cranioorbitozygomatic craniotomy. The individual surgical approach was chosen for tentorial and petroclival meningiomas. Lesions situated predominantly in the cerebellopontine angle were approached via retrosigmoid craniotomy or combined presigmoid–retrosigmoid craniotomies. In cases with tumour extension into both the middle and posterior fossae, we most often chose a combination of approaches through a two-staged resection or the combined petrosal approach. Our surgical strategy was based on maximal but, above all, safe meningioma resection. Gross total resection (GTR) was not attempted on tumours invading the cavernous sinus owing to high morbidity, and surgical resection of the intracavernous portions was never performed. Furthermore, for lesions infiltrating vital neurovascular structures of the skull base, aggressive tumour resection was also avoided. The extent of resection was determined based on surgical reports and assessments of 3-month follow-up MR imaging: near-total resection (NTR) was considered in cases of intracavernous residual tumours, and subtotal resection (STR) was defined as leaving an extracavernous residual tumour behind.

### 2.4. Postoperative Radiotherapy

Postoperative fractionated stereotactic radiotherapy (FSRT) or stereotactic radiosurgery (SRS) was applied in our cohort. Recommendations regarding adjuvant treatment with radiotherapy or radiosurgery were made by the consulting oncologist and were not uniform throughout the analysed period. Therefore, in groups of patients with a similar tumour resection extent, some were qualified for adjuvant treatment, while others were not. Patients with a postoperative KPS of less than 70% were not treated with postoperative radiotherapy or radiosurgery.

### 2.5. Parameters Assessed

The clinical, operative, and hospital records of the included patients were retrospectively reviewed. Patient demographics, neurologic findings, radiologic results, surgical details, tumour size, extent of resection, histopathologic features, the use of adjuvant radiation, and recurrence rates were recorded. Patients were examined at the outpatient clinic. Patient conditions were assessed at follow-up based on neurological examination and brain MRI. MRI scans were conducted on all patients preoperatively, 3 months postoperatively, and at subsequent regular 1-year intervals. Neurologic outcomes were assessed using the Karnofsky Performance Scale (KPS). Tumour progression was defined as a noticeable growth of residual tumour on follow-up MRI. The time to meningioma recurrence was determined from the date of surgery to the date of the first MRI scan showing tumour progression. Patients underwent follow-up until reaching a recurrence endpoint for progression-free survival (PFS) analysis. In patients who did not experience progression, follow-up was censored at the last MRI study.

### 2.6. Statistical Analysis

Statistical analysis was performed using Jamovi [3]. Counts and percentages for qualitative variables were presented to provide descriptive statistics. Interquartile ranges (IQRs) and medians were used to characterize quantitative variables. The differences between independent groups were analysed using the Kruskal–Wallis one-way analysis of variance test in cases of more than 2 groups; otherwise, the Mann–Whitney U test was applied for quantitative variables and Fisher’s exact test or Pearson’s Chi-squared test for qualitative variables. For multiple comparisons, the false discovery rate (FDR) approach was employed. For correlation analysis between quantitative variables, Spearman’s rank correlation coefficient was used. The null hypothesis, according to which there is no difference in survival between two independent groups, was tested using the log-rank test. The difference in survival times between independent groups was tested using Cox regression. If the assumption on proportionality of hazards was not met, then the coxphw package was applied using R. A 0.05 alpha level was used for all analyses.

## 3. Results

### 3.1. Patient Clinical Data

Between January 2006 and May 2020, 39 patients were operated on for parasellar meningioma invading the cavernous sinus. Two patients died in the early postoperative period, and five others were not included because of a lack of follow-up data. A total of 32 patients had a follow-up of at least three years, met the inclusion criteria, and were suitable for the tumour progression analysis.

The patient population consisted of 9 male (28%) and 23 female (72%) patients with a median age of 44 years (range, 22–68 years). There were 13 petroclival, 10 medial sphenoid wing, and 7 tentorial incisura meningiomas, and in two cases, the tumour was predominantly confined to the cavernous sinus. In nine cases (seven petroclival and two tentorial meningiomas), a staged resection was performed via retrosigmoid and pterional craniotomy. We identified 10 (31%) patients with NTR and 22 (69%) patients with STR. Meningiomas that were removed using NTR were significantly smaller than those for which STR was performed: the median tumour size for tumours subjected to NTR was 16.75 mm (median range, 8.1–26.4) compared with 21.10 mm (9.6–42.3) for meningiomas that underwent STR (*p* = 0.03). 

Postoperative radiotherapy was applied to 18 (51%) patients, more likely in patients with subtotal tumour resection (15 out of 22 after STR compared with 3 out of 10 after NTR, *p* = 0,04). Ten patients (31%) were treated with FSRT, including eight cases after STR and two cases of NTR. SRS was applied as an adjuvant therapeutic modality for residual tumours in seven patients after STR and one patient after NTR.

### 3.2. Intraoperative Observations

Apart from two cases of primary cavernous sinus meningiomas, all other patients exhibited secondary invasion of the cavernous sinus, associated with the extension of the meningioma into the cavernous sinus from a parasellar region. During surgery, the resection margin was defined by the wall of the cavernous sinus, when it could be identified, or by the onset of bleeding from the cavernous sinus. We assumed that the tumour within the cavernous sinus would not be removed, nor would decompression of the cavernous sinus be performed. Consequently, every resection involved leaving the intracavernous portion of the tumour intact. The distinction between NTR and STR was determined based on the extent of tumour infiltration into the vascular and neural structures beyond the cavernous sinus: in cases where safe tumour dissection was not feasible, the tumour was removed subtotally. No anatomical pattern of cavernous sinus invasion was observed that correlated with a greater or lesser extent of meningioma resection. The extent of resection was not related to the location of the meningeal attachment and depended solely on the preserved plane of dissection between the arachnoid and the tumour in the parasellar area.

### 3.3. Tumour Progression

**Figure 1:**
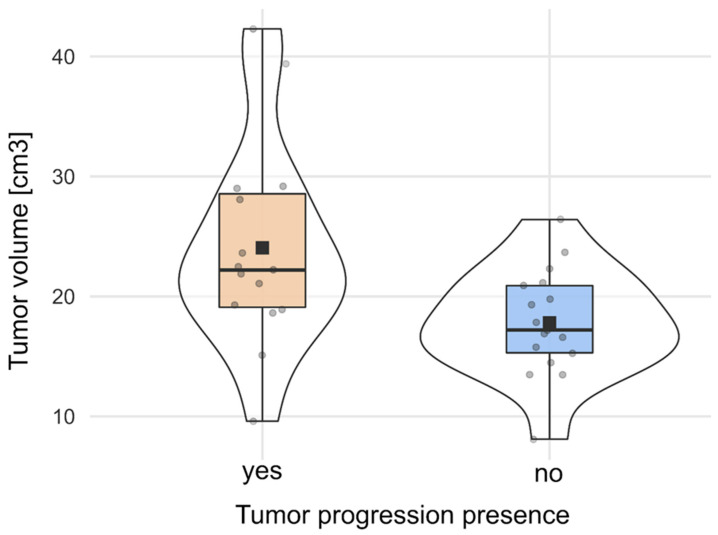
Tumour volume difference in relation to the presence of tumour progression.

The median radiological follow-up period for the entire cohort was 8 years (95% CI, 7.08–9.86; range, 3–17 years). During this period, radiological tumour progression was found in 15 patients (46.9%). Tumour progression was found significantly more often in patients with larger tumours and cases of marked brain oedema surrounding meningiomas. Patients with tumour progression had a median tumour volume of 22.20 cm^3^ (95% CI, 19.32–28.79) compared with 17.20 cm^3^ (95% CI, 15.54–20.07) in patients with stable tumours (*p* = 0.016) (Figure 1). Tumour progression occurred in 12 of the 18 cases (67%) of brain oedema seen on preoperative MRI, but it was found in only 3 of 14 cases (21%) without this finding (*p* = 0.011). Although tumour progression after near-total resection was observed less frequently (20%) than in patients who had STR (59%), the difference did not reach statistical significance (*p* = 0.06). Table 1 summarizes cohort characteristics in relation to meningioma progression.

### 3.4. Long-Term Outcomes in the Kaplan–Meier Survival Analysis

**Figure 2:**
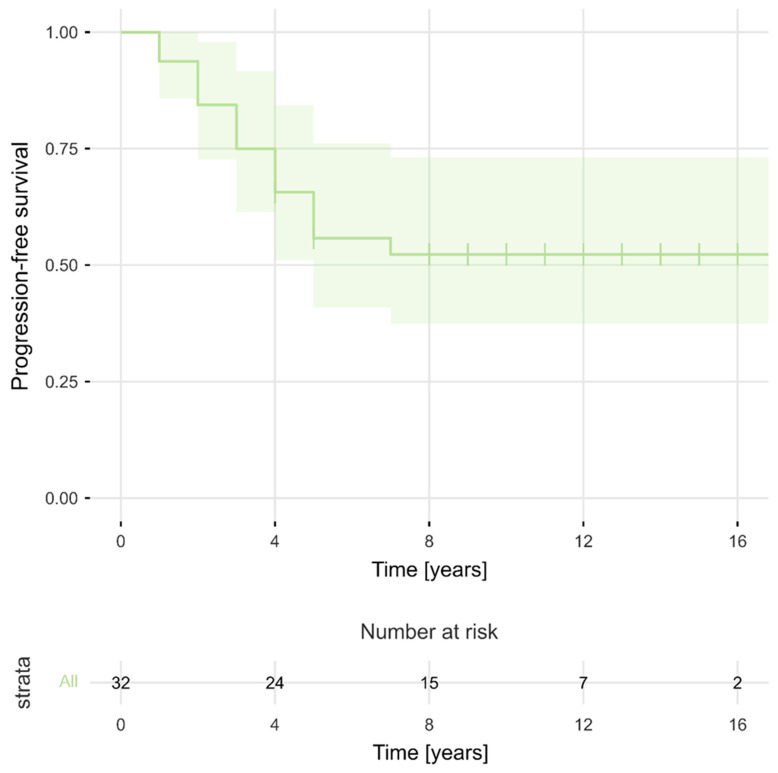
Progression-free survival for the entire cohort of 32 patients.

**Figure 3:**
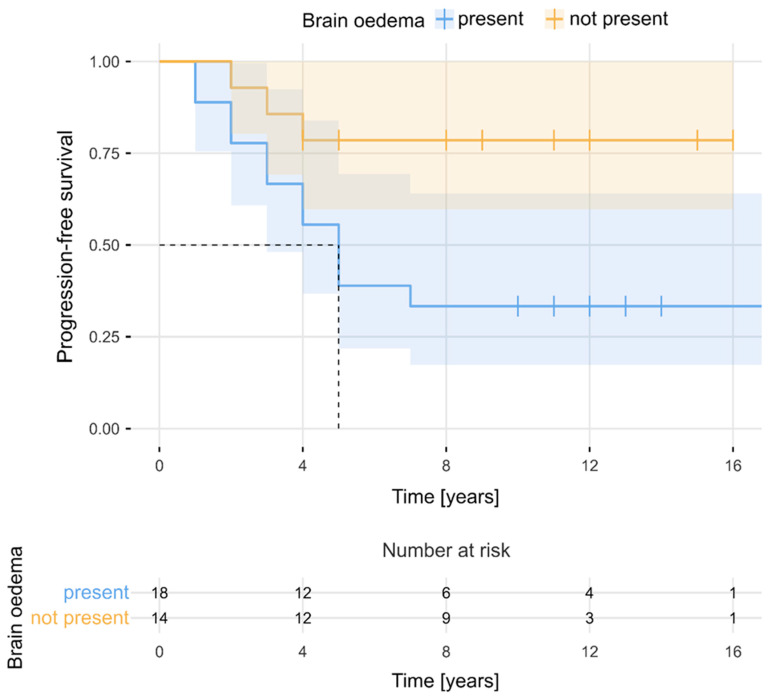
Progression-free survival in relation to the presence of brain oedema.

**Figure 4:**
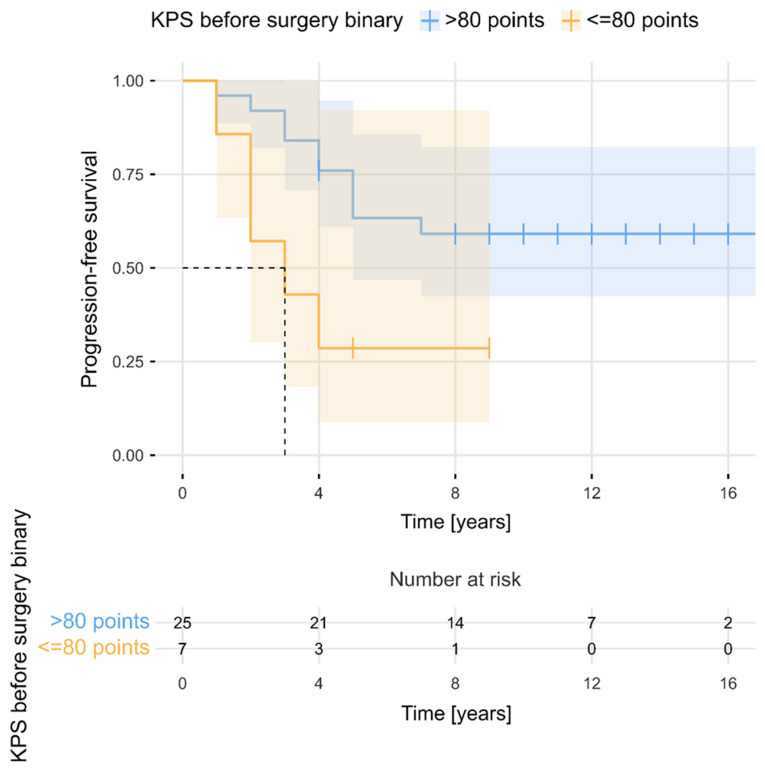
Progression-free survival in relation to the dichotomized KPS score before surgery.

**Figure 5:**
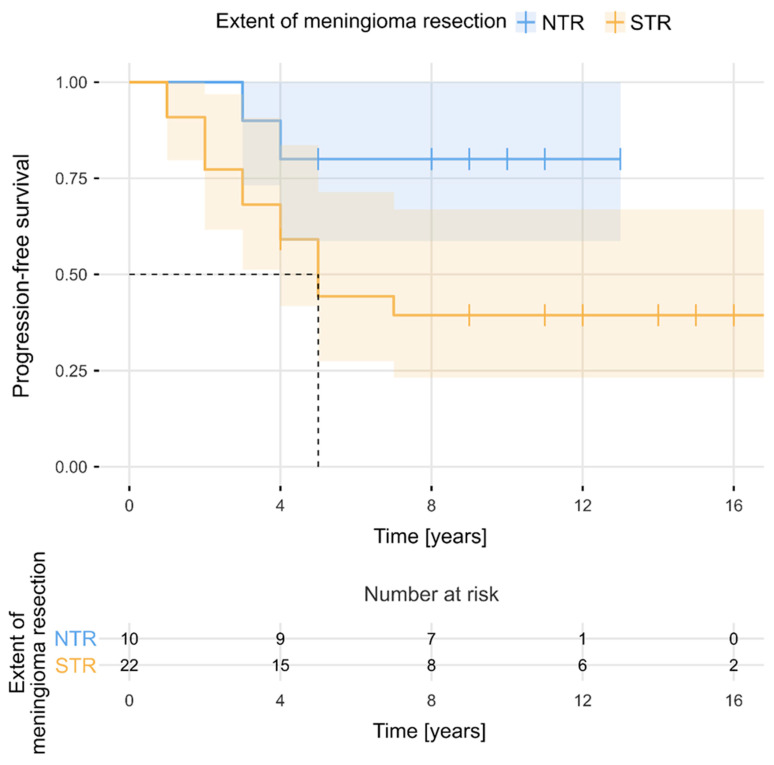
Progression-free survival in relation to the extent of meningioma resection.

**Figure 6:**
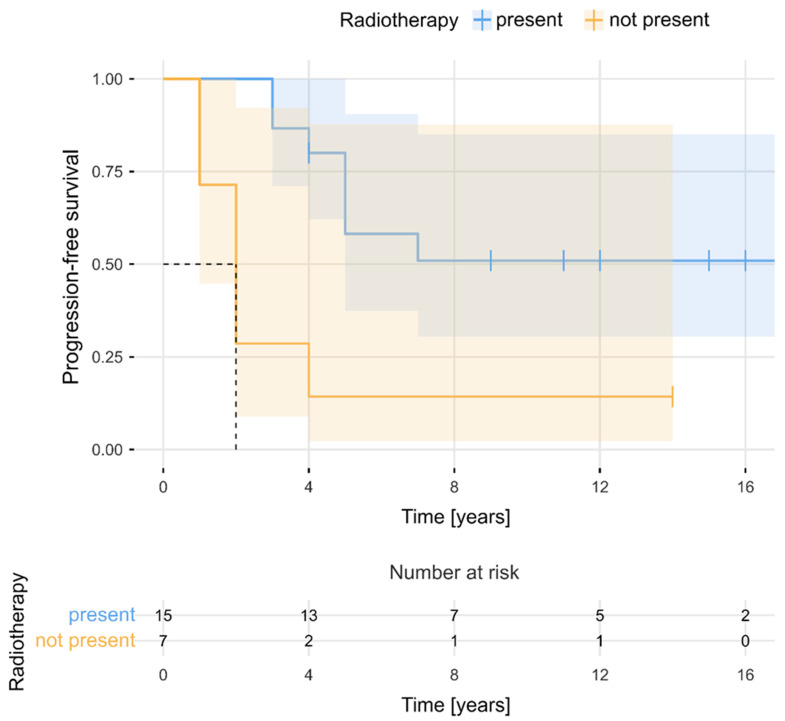
Progression-free survival in relation to the presence of radiotherapy.

The estimated median PFS period for the entire cohort was 11 years (95% CI, 9–14). PFS rates at 2, 3, 5, 7, and 10 years after the surgery were 84%, 75%, 56%, 52%, and 52%, respectively. Figure 2 presents PFS for the total sample (N = 32). As can be observed, PFS became stable after 7 years. The 5-year number of progressions and 5-year PFS rates were determined separately in relation to selected clinical factors. The Kaplan–Meier survival curves (Figure 3 and Figure 4) showed that evidence of brain oedema and a neurological condition equal to or less than 80 KPS were associated with unfavourable PFS (log-rank tests, *p* = 0.021 and *p* = 0.034, respectively). PFS over the follow-up period did not differ significantly in relation to sex, tumour location, use of postoperative radiotherapy, or even the extent of meningioma resection. Patients who underwent NTR had 5-year PFS rates of 80% compared with 44% 5-year PFS for patients treated with STR (log-rank test, *p* = 0.051) (Figure 5). There was also a non-significant difference between the Kaplan–Meier PFS curves showing that patients with larger tumour volumes (>20 cm^3^) had worse PFS (*p* = 0.053). Interestingly, the Kaplan–Meier curves did not differ between patients who received postoperative radiotherapy and those who did not qualify for adjuvant treatment (5-year PFS values of 60% and 50%, respectively; log-rank test, *p* = 0.403). However, patients with subtotal tumour removal progressed less often after radiotherapy (7 out of 15, 47%) than those without radiotherapy (6 out of 7, 86%), and Kaplan–Meier survival curves differed significantly in these groups (5-year PFS, 58% vs. 14%; log-rank test, *p* = 0.003) (Figure 6).

### 3.5. Factors Affecting Meningioma Progression in Cox Regression Analysis

Logistic regression analyses were used to prognosticate significant factors for tumour progression. The association of age, tumour volume, and KPS score with meningioma progression was analysed in both continuous variable and binary variable models (Table 2 and Table 3). A univariate analysis based on a continuous variable model showed that the risk of meningioma progression after surgery increases along with its size in preoperative measurements (*p* = 0.001) and worse neurological conditions (according to KPS) before surgery (*p* = 0.003). The analysis performed on the binary variable models confirmed that patients with lower KPS scores progressed more frequently (*p* = 0.042). We also found that marked brain oedema surrounding the tumour significantly affected meningioma progression (*p* = 0.034). The brain oedema and tumour size factors were subsequently analysed in the multivariate model, which demonstrated that tumour size was an independent factor in meningioma progression after surgery (*p* = 0.012) (Table 4).

### 3.6. Neurological Outcome

**Figure 7:**
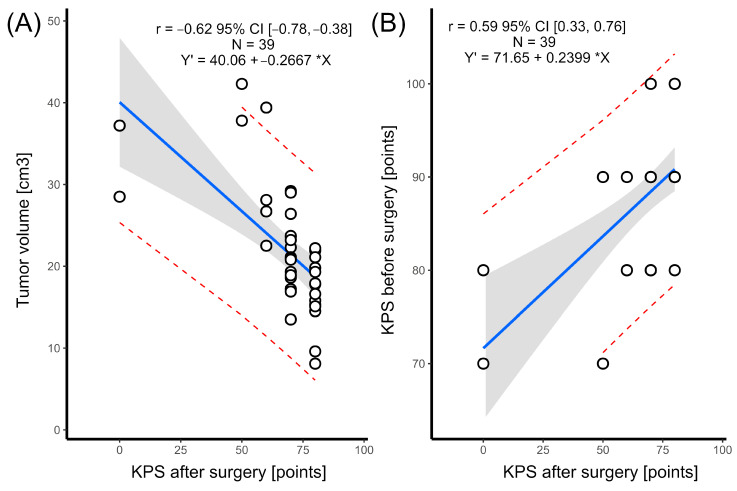
Relation between tumour volume and KPS after surgery (panel (**A**)) and between KPS before surgery and KPS after surgery (panel (**B**)). Regression lines are blue, grey areas denote confidence interval, while red dashed lines denote prediction intervals.

**Figure 8:**
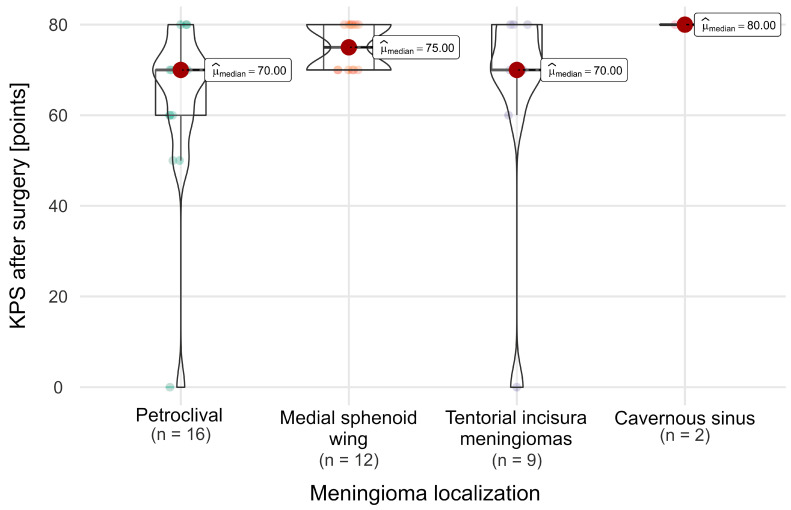
KPS after surgery score difference in relation to meningioma localization.

Considering all 39 operated patients with parasellar meningioma invading the cavernous sinus, we analysed the surgical treatment results, with particular emphasis on the functional outcomes. The median preoperative Karnofsky Performance Scale (KPS pre-op) was 90% (95% CI, 86.58–91.55), and the median postoperative KPS (KPS post-op) was 70% (95% CI, 69.75–75.25). We found that a patient’s good postoperative neurological condition (KPS of 80 or more) was significantly correlated with a smaller tumour volume (*p* < 0.001) (Figure 7A), a better preoperative functional state (*p* < 0.001) (Figure 7B), and meningioma localization on the skull base, with petroclival and tentorial incisura tumour patients having lower postoperative KPS scores (*p* = 0.045) (Figure 8).

## 4. Discussion

This publication presents the long-term results of treating parasellar meningiomas invading the cavernous sinus, with particular emphasis on the risk of tumour progression at the postoperative follow-up. Meningiomas in this location are confined to the cavernous sinus or, more frequently, invade it secondarily from adjacent regions, such as the medial sphenoid wing, the petroclival region, or the tentorial incisura [2,4].

Cavernous sinus invasion makes complete meningioma removal a hazardous treatment associated with a very high rate of morbidity and death owing to the risk of damage to the internal carotid artery and cranial nerves. Thus, complete safe resection is very rarely possible, and adjuvant stereotactic radiotherapy or radiosurgery is usually considered. Using radiotherapy as part of the multimodality management of skull base meningiomas has become extremely popular in the last two decades. However, it should be noted that radiotherapy and radiosurgery are often limited for larger tumours with extracavernous extension, and their effectiveness in reducing the mass effect is low [5]. Moreover, the close relationship between the tumour and other important neurovascular structures carries an additional risk of severe neurological impairment [6,7,8]. The EANS Skull Base Section [9] recommends surgical resection to relieve mass effects, leaving tumour remnants to adjuvant therapy whenever necessary. This requires close radiological surveillance of the residual tumour, and adjuvant radiotherapy is initiated upon the first evidence of tumour growth. However, it is to be determined whether radiotherapy/radiosurgery should be applied right after surgery or whenever residual tumour growth is observed during the follow-up. On the one hand, the risk of meningioma progression may be reduced with early radiation, but on the other hand, the risks associated with radiotherapy must be taken into account concerning cranial nerve function [10]. Experience indicates that only some cavernous sinus meningiomas grow during long-term follow-up [11,12]; however, given their rarity, our knowledge of the nature of these tumours is based only on small case series and should be interpreted with some care. Hence, our observations may contribute to knowledge in this field and allow us to establish rational treatments.

The most striking observation in the analysed group of patients was postoperative radiotherapy’s lack of influence on the risk of tumour progression for the entire cohort. Radiotherapy significantly reduced the risk of tumour progression only after the subtotal removal of the tumour, wherein tumour remnants are left outside the cavernous sinus. Notably, despite the predominantly subtotal resection of meningiomas invading the cavernous sinus, half of these tumours remained stable over long-term follow-up, whether irradiated or not, and no tumour growth was observed beyond 7 years post-surgery. This may confirm the observations of other authors showing that meningioma remnants left within the cavernous sinus are often indolent tumours; the risk of progression is low, and attentively monitoring the residual mass is appropriate [13]. The effectiveness attributed to radiotherapy in stabilizing tumour growth might be the result of the natural history of these benign tumours, which suggests we should use a more cautious approach and avoid unnecessary irradiation. Amelot et al. [12] studied the natural history of these tumours in a prospective series of 53 consecutive patients suffering from meningiomas strictly confined to the cavernous sinus; they found that simple symptomatic treatment (short courses of corticosteroids and carbamazepine) allowed most patients to become asymptomatic. All patients with incidental findings remained asymptomatic, and 83% of tumours did not show any significant growth at the end of the median follow-up of 10.2 years. Furthermore, only 9% of patients required additional radiation therapy. Ehresman et al. [13] observed sinus meningioma growth rates and found that tumours outside the sinuses exhibited nearly twice the average growth rate as tumours inside the sinuses. Their study confirmed that although GTR is always the most optimal treatment for meningiomas, the further growth of the tumour remnants left inside the sinus may not be as robust as expected. In a series of 105 cases of medial sphenoid wing meningiomas involving the cavernous sinus, Masalha et al. [14] found that maximal safe resection and postoperative stereotactic radiotherapy for the residual tumour significantly reduce the risk of tumour progression. By contrast, Gozal et al. [15] reported that patients treated with and without adjuvant radiation demonstrated similar actuarial tumour control rates at 5 years. Although the importance of resection extent regarding the risk of meningioma progression seems obvious, published case series on cavernous sinus meningiomas provide ambiguous conclusions. Masalha et al. [14] and Mathiesen et al. [16] reported that recurrence was significantly more frequent after STR, while other studies have reported minimal difference or similar recurrence rates after STR and GTR [2,17,18].

Our study supports the importance of radical extracavernous tumour resection for achieving satisfactory tumour growth control, as the extent of meningioma resection influenced PFS in this series. We believe that radiation therapy has its place as second-line therapy when an extracavernous tumour remnant is left after surgery or in cases of intracavernous tumours showing progress in follow-up radiological controls. In cases of subtotal tumour resection, we observed that the subsequent use of adjuvant radiotherapy or radiosurgery significantly reduced the risk for further meningioma progression. We also found that brain oedema and tumour size had significant impacts on meningioma progression in the long-term follow-up, which is probably associated with the extent of tumour resection. Only two patients in our cohort had tumours confined to the cavernous sinus. Other meningiomas in this series were different in origin, and many were large tumours with great potential to infiltrate critical neurovascular structures outside the cavernous sinus. These tumours were frequently subtotally resected, which increased the risk for further progression. Furthermore, a greater tumour growth rate and marked peritumoural brain oedema may indicate more aggressive meningioma behaviour and a higher risk of recurrence. These factors—as well as younger age at diagnosis, a lack of calcification on radiological scans, menopause, and the presence of symptoms—are associated with increased meningioma growth rates and have been identified previously [19,20,21,22]. 

In our study, postoperative good functional outcomes were not affected by the extent of tumour resection but were significantly correlated with better preoperative functional states and a smaller preoperative tumour volume. In 2016, Nanda et al. [17] demonstrated improvement in 75% of patients with CN III–VI dysfunction and 54% of patients with visual deficits in a cohort of 65 patients treated with varying degrees of surgical removal. In this study, 54% of patients had new cranial nerve deficits, ultimately resolving in 62.5% of those cases. Morisako et al. [23] found that a greater resection extent was associated with more perioperative complications. By contrast, in a case series of 27 patients with sphenopetroclival meningiomas, Martínez-Pérez et al. [24] showed that a higher resection extent is not necessarily associated with a higher incidence of surgical complications. In their study, when the plane between the tumour and other important neurovascular structures was poor, a thin sleeve of residual tumour was left in place to preserve neurological function. As a result, patients with small residual tumours had better functional outcomes at follow-up. These studies highlight the importance of the cavernous sinus and optic nerve decompression for functional recovery. Our strategy also assumed a constant attempt to reach a maximal safe resection to reduce postoperative tumour volume and the risk of postoperative neurological deficits by relieving mass effects on the surrounding structures [15,25,26]. 

## 5. Limitations of the Study

The main limitation of this study is its retrospective design and the analysis of patients operated on at a single centre. Although the vast majority of surgeries were performed by a single neurosurgeon, assessing the extent of resection—including the degree of invasion in the cavernous sinus and the vascular and neural structures beyond the sinus—remains subjective and needs to be taken into account when interpreting the results. The postoperative management strategy varied between cases. As noted in the methodology section, the criteria for adjuvant radiotherapy were not uniform, and patients with similar tumour resection extents received either adjuvant radiotherapy or were only observed. We should also emphasize that the study group was heterogeneous, including skull base meningiomas in different parasellar locations and significantly varied tumour sizes. Finally, a larger study group and a longer postoperative follow-up period might have yielded more statistically significant results.

## 6. Conclusions

Although an ideal treatment strategy for symptomatic parasellar meningiomas with intracavernous invasion was not established, our study provides evidence for safe extracavernous tumour removal strategies for brain and neural decompression. When leaving a tumour component outside of the cavernous sinus, we recommend using adjuvant stereotactic radiotherapy or radiosurgery to achieve tumour control (depending on the tumour’s proximity to the optic apparatus). For residual intracavernous meningioma remnants, we propose that close radiological surveillance and radiotherapy only have to be initiated if there is evidence of further tumour growth. Based on the stable PFS in the long-term follow-up, we recommend annual MRI follow-ups for all patients during the first 7 years post-surgery. Subsequently, for stable tumours, a follow-up MRI can be performed every 2 years.

## Figures and Tables

**Table 1:** Patient and tumour characteristics according to tumour progression.

Characteristic		No. of Patients	Any Meningioma Progression	No Meningioma Progression	*p*-Value
Sex	Famale	23	12	11	0.44
	Male	9	3	6	
Age (yrs) median (95% CI)		32	44 (36.85–50.42)	41 (39.39–48.79)	0.95
Tumor location	Petroclival	13	7	6	0.95
	Sphenoid wing	10	4	6	
	Tentorial incisura	7	3	4	
	Cavernous sinus	2	1	1	
Tumor volume * (cm^3^), median (95% CI)		32	22.20 (19.32–28.79)	17.20 (15.54–20.07)	0.016
Brain oedema *	Yes	18	12	6	0.011
	No	14	3	11	
Extent of surgery	NTR	10	2	8	0.06
	STR	22	13	9	
KPS pre-op	70	1	1	0	0.069
	80	6	4	2	
	90	20	10	10	
	100	5	0	5	
Post-op FSRT/SRS	Yes	18	8	10	0.75
	No	14	7	7	

NTR—near total resection, STR—subtotal resection, KPS pre-op—preoperative Karnofsky Performance Scale outcome, Post-op—postoperative, FSRT—fractionated stereotactic radiotherapy, SRS—stereotactive radiosurgery, *—in the preoperative MRI.

**Table 2:** Univariate analysis (continuous variable model) for PFS in patients with cavernous sinus meningiomas.

Characteristic		All	Univariate Analysis	
			HR (95% CI)	*p*-Value
Age	Mean (SD)	44.0 (9.9)	1.01 (0.95–1.06)	0.817
Tumor volume * (cm^3^)	Mean (SD)	20.7 (7.3)	1.13 (1.05–1.21)	0.001
KPS pre-op (pts)	70	1 (3.1)	0.89 (0.82–0.96)	0.003
	80	6 (18.8)		
	90	20 (62.5)		
	100	5 (15.6)		

PFS—progression-free survival, SD—standard deviation, KPS pre-op—preoperative Karnofsky Performance Scale outcome, *—in the preoperative MRI.

**Table 3:** Univariate analysis (binary variable model) for PFS in patients with cavernous sinus meningiomas.

Characteristic		Univariate Analysis	
		HR (95% CI)	*p*-Value
Age	>40	1	0.943
	≤40	1.04 (0.37–2.92)	
Tumor volume * (cm^3^)	>20	1	0.065
	≤20	0.36 (0.12–1.06)	
Brain oedema *	Yes	1	0.034
	No	0.25 (0.07–0.9)	
Extent of resection	NTR	1	0.073
	STR	3.92 (0.88–17.40)	
KPS pre-op	>80	1	0.042
	≤80	3.10 (1.04–9.23)	
Post-op FSRT/SRS	Yes	1	0.416
	No	1.53 (0.55–4.22)	

PFS—progression-free survival, NTR—near total resection, STR—subtotal resection, KPS pre-op—preoperative Karnofsky Performance Scale outcome, Post-op—postoperative, FSRT—fractionated stereotactic radiotherapy, SRS—stereotactic radiosurgery, *—in the preoperative MRI.

**Table 4:** Multivariate analyses for PFS in patients with cavernous sinus meningiomas.

Independent Factor		All	Univariate Analysis		Multivariate Analysis	
			HR (95% CI)	*p*-Value	HR (95% CI)	*p*-Value
Tumor volume * (cm^3^)	Mean (SD)	20.7 (7.3)	1.13 (1.05–1.21)	0.001	1.11 (1.02–1.2)	0.012
Brain oedema *	Yes	18 (56.2)	1	0.034	1	0.189
	No	14 (43.8)	0.25 (0.07–0.9)		0.41 (0.11–1.55)	

PFS—progression-free survival, SD—standard deviation, * in the preoperative MRI.

## Data Availability

The original contributions presented in the study are included in the article, further inquiries can be directed to the corresponding author/s.
